# Buffalo Academy of Medicine

**Published:** 1911-06

**Authors:** 


### Buffalo Academy of Medicine

A SPECIAL memorial meeting of the Buffalo Academy of Medicine was held May 2, to take action on the death of Dr. Carlton C. Frederick, its president. The memorial adopted is printed elsewhere. Addresses were made as follows:

Dr. Lothrop in addressing the Academy said:

After an intimate association of twenty years words are inadequate to express the feeling one has toward the man who has been to him a teacher, friend, a good, great, guiding brother. I feel in the loss of Dr. Frederick the loss of one nearer than a friend. I feel the loss of a brother. He has been to me first, a teacher and always a teacher, but mainly and largely the sort of friend of whom my father used to speak; "one who would stand without hitching." Of the personal side of his character I learned much in my early days as a student. His popularity among the boys in his class in obstetrics was striking, a popularity that was obviously due to the fact that he was absolutely unselfish with the knowledge he possessed. It seemed to me that he had a personal interest in every member of his class; that it was his desire to give everyone information on any subject which he knew anything about. That attitude was not confined to the class room but his students were welcome to come to his office at any hour of the day or night to have their problems solved with his assistance. In addition to his duties as teacher and lecturer he made us cordially welcome to any clinic, case or exhibition of medical interest with which he was connected. I think this characteristic of absolute unselfishness stamped him at the beginning of his teaching career.

His early days at the hospital I recall as though it were yesterday. The first laparatomy, a double pyosalpynx, under the guidance of Dr. W. S. Tremaine, and the succeeding hardships and struggles to which he was subjected in his efforts to adapt antiseptic surgery to the needs of the individual. It was the good old days, you know, when we washed our hands in pus and then operated; those days when wounds not full of pus were not healthy wounds.

I have witnessed the untiring patience with which he sought to unravel the mysteries of his art, the kindness he exhibited toward his patient, his forgetfulness of self, his willingness to stand at the bed-side if he felt his mere presence would comfort the patient; and those characteristics of Dr. Frederick persisted until his last breath. He was absolutely devoted to his work, to his patients and their interests; no effort was too great; it was no trouble to do anything. When he came down in the morning if he knew some patient was blue, depressed or doubtful as to the outcome of her trouble he always seemed to remember to bring a flower, bunch of flowers, a plant or perhaps a package of chewing gum—some little trinket that would appeal to the individual who was down and out; something to brace her up. That characteristic exhibited itself in the early days of the hospital and persisted throughout his life in treating the many thousands of people who have passed through his hands.

This same feeling of generosity and unselfishness was extended to his coworkers and his nurses. All who served as his assistants or who have nursed under him cherish the warmest feeling toward the personal side of Dr. Frederick as well as the keenest admiration for the professional side. In my experience as his assistant, I always felt I had some one to whom I could go and confess my ignorance and my mistakes, some one who would pat me on the back and say: "Well, old man, never mind I have done it a hundred times myself, you will not do it again. I have had this same experience and we will work it out together." The young doctors who made mistakes he would take aside and say: "Now you have made a mistake, but we will work it out all right."

Of his geniality and hospitality we have heard much. I feel almost as though the last word had been said on that characteristic which was so predominant. One point might be mentioned, his love of the beautiful. I mean he had an artistic sense which manifested itself in his love of flowers, of nature, of works of art, of oriental rugs, of which he was an excellent judge, having read nearly all the literature on the subject and having a very intimate association with dealers whereby he learned many of the finer points which most of us miss.

As a traveling companion one could hardly fail to appreciate the keen sense of humor which he possessed. I think the pleasantest periods of our association were the trips we took, for in the early days we often went away together. If some investigation seemed promising he went to find out about it and always took me with him. The last few years of his life his spirit of unselfishness manifested itself in a stern sense of professional duty. We, who knew the state of his physical forces, endeavored to assist him and to take from him this overload which he had no business to carry. But his sense of duty was so strict, that no matter what time a call came if he felt they wanted him he would sooner sacrifice himself than send an alternate who might prove to be unsatisfactory. I think this overstrain of the last few years was responsible for his break down.

In the last few days of his life, when this condition developed, he was keenly conscious of the fact that it was the end. Only within a week he had expressed his opinion as to the nature of his trouble and it was found at the autopsy. During the last few days he was cheerful, manifesting the greatest confidence in those about him, feeling that all was right.

His last words, as the breath was leaving his body, spoken to those immediately around him, were: "Dilatation? Yes. Good-bye." I think when all is over, and the last tribute is paid, there will always remain a memory of Frederick as a surgeon, a gentlemen, a friend.

Dr. Pryor said:

By the untimely death of Dr. Frederick I have been bereft of one of my best and warmest friends. I knew him as a young man for I was his assistant in the General Hospital. Here began a friendship that lasted until his death. As his assistant I can never forget his kindness, his thoughtfulness, his consideration for me.

He had certain characteristics which I regard as very remarkable; he was one of the most tireless workers I have ever known; there seemed to be no limit to his energy. He would toil early and late. He had other characteristics which marked him for success in life. He had temperament, character, mind, bodily strength and endurance. One of the peculiar things in regard to him and which was most noticeable was his prompt and early inclination for the line of specialism which he later followed. His whole mind seemed bent toward that particular department of medicine. Later, after leaving the hospital, he gradually began his work in surgery and, like many other men who have reached his age, his career was made peculiar in that it marked the beginning of aseptic surgery. While we were internes in the General Hospital the first Lister dressing was made in that institution. One of our physicians while abroad had observed antiseptic work and returning to Buffalo made the first Lister dressing in this city.

I have never known a man in the medical profession more thoroughly devoted, or whose life was more consecrated, to his work. There was nothing he would not do for a fellow practitioner. He would respond early or late and was never too tired to help him. He probably did as much for charity as any physician or surgeon in this city. How he stood the strain as long as he did has always been a mystery to me.

He was handicapped to a certain extent by disease but paid no attention to it. There seemed to be no time when he would not work or go any place at any hour. I regard him as a remarkable example to the younger men of the profession because he belonged in a way to the old time school of physicians that seemed to a certain extent to be disappearing.

He had a most charming and happy disposition and was a good comrade. He had feeling and kindly thought for his patients and they loved him. Here, tonight, I personally acknowledge many debts of gratitude when he was of the greatest assistance to me in my work. He was in many ways an ideal consultant having skill, experience, common-sense and judgment. He was not simply an operator but a surgeon.

You will remember, all of you, a certain quotation from Lowell's Commemoration Ode:

"Our slender life runs rippling by and glides
Into the silent hollows of the past;
What is there that abides
To make the next age better than the last!
Is earth too poor to give us
Something to live for that shall outlive us?"

In my opinion Dr. Frederick answered that query excellently in the affirmative. He prevented and pushed aside much suffering, much woe. Many, many people are happier for his existence and are grateful to him today. He was in many ways what I consider the ideal physician.

Dr. Mann spoke as follows:

I would like to say a few words in regard to Dr. Frederick. As has been already stated there is for many of us a sense of very keen personal loss in this event and I am sure I feel it because Dr. Frederick and I always have been the best of friends from the time he was interne in the General Hospital. When I began my duties there he was my assistant. After he left the hospital he continued to be my assistant until Dr. Lothrop made him offers which I advised him to accept. During the unfortunate controversy which followed the founding of Niagara University Medical Department he went to the other side, but our friendship was never disturbed. When the colleges were united it gave me the greatest pleasure to nominate him for clinical professor of obstetrics. When we met it was always a very great pleasure to me for we always found so many things of common interest; found so much to talk about. If we tired of medicine we could turn to flowers in which he was an adept.

I shall always feel it a very great sorrow that Dr. Frederick went before I did because he was a good deal younger man than I am. He was something more than a mere friend and acquaintance. He was entitled to be termed one of the great men of Buffalo; he was one of the beginners, one of the founders, of the science and art of gynecology. He was one of the men to do the earlier work here and his reputation extended over all the country.

His skill has been alluded to. Strangely enough I have never seen him work, but I have heard from others that he was a most skilful operator. His results I have seen and they were exceedingly good. We cannot afford to lose such men for there are not many of them; and who are to take their places it is hard to say. They do not come everyday.

I know this Academy of Medicine will express well a very deep loss which the profession feels as a whole. It is a misfortune and I am sure I only express the feelings of all when I say I am exceedingly sorry. I do not, I cannot say anything more.

---

Dr. Hayd paid the following tribute to Dr. Frederick's memory:

Tonight we are called together to express our profound grief over the death of our friend and colleague, Dr. Carlton C. Frederick. About thirty years ago, when I came to Buffalo, I first met Dr. Frederick and early in my career I was on terms of intimate friendship with him and during all those years of close contact and professional association my affection and interest grew deeper, so that I was always able to number him among my warmest and closest friends. Living, as we did, in the same city, and having ambitions along the same lines of practice and stimulated into competitive fields of labor by the same hopes of possible rewards, I was given the very best opportunities to know Dr. Frederick's true value as friend, rival and ever-willing counsellor and especially so because our business interests often clashed: "he got my cases and I got his." My first hospital appointment as gynecologist I held with him at the Erie County Hospital, and my private work for many years was done at the Woman's Hospital, where he always stood ready, with cheerful heart and hand, to help and make easy my first operative undertakings. After a few years of general practice our interests developed along the same lines of work and both of us became members of the American Association of Obstetricians and Gynecologists, he being my senior by four years, and on behalf of that distinguished body, I beg to present this brief and inadequate memorial:

In 1891 Dr. Frederick became an active member and from his initiation took a great interest in the work of the association and presented a paper—often annually—upon different practical questions which were engaging the attention of the active surgeons, and at the same time took part in various discussions. His first paper was a plea for conservative operations upon the appendages, and in that paper he vigorously protested against the removal of the ovaries for purely functional troubles—so-called reflex disturbances—unless gross pathology could be made out by vaginal and pelvic examination. His contributions to our transactions are many and his papers are usually devoted to the consideration of some live subject, as neurasthenia accompanying and simulating pelvic disease, "which is the preferable operative method for holding the uterus in position?" "Some rare and odd cases and experiences in pelvic and abdominal surgery and the lessons they teach," and so on. Perhaps, no paper gave him the national reputation, and showed the wealth of material and the amount of experience at his command, as did that one he wrote upon the degenerations which take place in uterine fibroids. These observations attracted considerable attention and the deductions made in this paper stimulated a greater interest in his subject, and his statistics were quoted and commented upon by many writers in this important field of work. As a member of our association he became distinguished and men listened to what he had to say, because it represented advanced work in pelvic and abdominal surgery, and, secondly, because he was a practical, thoroughly honest, conscientious man, and of good judgment. His personal characteristics and sweetness of temper and suavity of manner brought to him many close relations in the society. Men looked upon him as a fair and honorable colleague. He possessed the happy faculty of being forceful, but yet considerate of the opinions and failings of others. He was fearless but not tyrannical. He was kind but not obsequious. He was self-appreciative and reliant but not conceited and boastful, and in debate and scientific discussion he was temperate, persuasive, practical and resourceful. Unfortunately, at the height of his influence when only fifty-six years of age, when the suffering and afflicted could most have benefited from his trained mind and experienced hand, and when the young surgeon could have called upon a responsive and sympathetic friend and helper in times of worry and distress, Dr. Frederick is suddenly and prematurely taken from us.

"And such is human life: so gliding on,
It glimmers like a meteor, and is gone."

---

Dr. Putnam's tribute was as follows:

Gentlemen, Dr. Frederick left the General Hospital the day I went in as interne and from that time to the present we were closely associated by professional bonds and by ties of friendship.

This meeting tonight is one which is especially his due; and it is good for us that we should assemble here to pay tribute to his memory. It is a good thing for us that the time has come when we lay aside all thought of self, forget our professional duties and meet to speak of the qualities of a man which have endeared him to so many. What Dr. Pryor has said is absolutely true; he was a consecrated man. It is sad that the profession of medicine is a jealous mistress who brooks no trifling, and in Dr. Frederick's case he gave his whole time, energy and his best work to the furtherance of that branch of medicine which he had adopted. He never was a man who did slighting work; he never was a man who refused the call of the poor; he was a man who was always at service of the families of his professional brethren and gave freely of his skill in accordance with those tenets of the profession which we all hold so dear and was glad to feel that whatever was in him was free for the benefit of all his fellow-practitioners. He not only helped them in their families but was generous in his consultations. He did much to educate other men in the line of surgery.

He will be missed by us; he will be very much missed by the profession not only in Buffalo but in the surrounding country; not only in this state but in neighboring states. He will be missed in scientific bodies for his contributions were always carefully prepared and are of permanent value. He will be missed by the laity to whom his skill brought so much of health, so much of comfort, to so many men.

I am very glad that I am here tonight and have been permitted to add my feeble tribute to the love and esteem in which we hold his memory.

---

Dr. C. A. Wall said:

I wish to say a word of the close relationship that has existed between Dr. Frederick and myself.

Of all the men who have spoken, who have known him, I antedate them all or almost all of them by ten years. We were schoolmates, Frederick and I and B. H. Grove, Frank Lewis and Steve Howell. We graduated from the high school together. But he and I had been comrades for many years before that and from then until the present time there had never occurred a single word of misunderstanding. Our social relationships have been most intimate from the time I first knew him. As boys we were together most of the time and I can most heartily endorse all that has been said about his kindly character and the consideration which he showed to all. It is a terrible loss which I feel tonight; it is hard for me to speak of him, he was so close a friend. I shall ever remember his unfailing kindness in all our associations of over forty years of intimacy.

Dr. S. Y. Howell's address was as follows:

I feel hardly equal to add to the mead of praise to which we have already listened. When medical men who are accustomed to death and who are, as the laity say of us, rather callous regarding the whole question of transmission to the life to come get together in this spontaneous way voice the praises of a man who has passed from us, it shows they are very deeply touched. It seems to me almost incredible. When on March 28 a number of us were together in honor of Robb, of Cleveland, Dr. Frederick was there, the life of the party, apparently in full enjoyment of health; and here four weeks afterward we are paying tribute to his memory. It simply exemplifies the chances and changes of this mortal life.

I have seen him once since that evening dinner. We were discussing in a jolly way automobiles in general and he was speaking enthusiastically of his electric and its advantages. There was then no premonition of this sudden end and to me it came as a frightful shock. I do not know as I have ever felt so deeply the death of a friend. I had been more or less associated with him throughout his life. It was equaled by Dr. Wall who was a member of his class in the Central high school where we graduated in 1873. Our paths then diverged. He went to Michigan and I to Columbia. We took our degrees in the same year. He took up medicine here, I went to the Physicians and Surgeons. After beginning our professional careers we were associated more or less, pursuing the same line of work.

He was one of the most genial, lovable men I have ever known. He was always a good fellow, always willing to help his friends. Whenever I was in need of hospital accommodations and could not get them his institution was thrown open to me and his assistance always volunteered in my behalf. His loss to me is terrible.

Some six or eight years ago I was called upon to examine him for the Mutual Life and finding his heart lesion was forced to say no to his application. I told him at the time that he ought to spare himself and avoid hard work. He seemed never to have taken the lesson to heart and, as his work increased, he seemed impelled to do all that was offered. He would do three or four operations in Buffalo and in the evening, when one should naturally enjoy the comforts of home, he would receive a telegram from an adjoining city and would start off and do another operation that night often working at midnight in some farmhouse by the light of a kerosene lamp, or candles, a task that is a heartbreaking strain, a heroic work, a terrific strain on his vitality and which undoubtedly told on that disabled heart. On that account his life was terminated much sooner than might otherwise have been the case. It seems a great pity that a man urged on by the sense of duty to do his work while there is yet time should be goaded on to sacrifice himself on the altar of duty. It is pitiful to think that this poor fellow might have lived many years if he had taken care of his poor, overtaxed heart. He did his duty faithfully and the end came prematurely. I sometimes think it is a better way after all, to be guided only by our own personal feelings, meeting the demands of life as they come, fighting on bravely, doing our duty while we live even though our span of life may be short. That is a question which may never be solved depending as it does on the personal equation. Carlton Frederick was able to do his work as he found it and when his end came, to lie down without fear or trembling to his last sleep, and may God bless him.

---

Dr. Charles G. Stockton's address was as follows:

I regret that an unavoidable absence from the city will prevent my taking part in the special meeting to be held this evening in memory of Dr. Carlton C. Frederick.

For many years I was closely associated with Dr. Frederick, both in personal and professional ways, and very strong ties were formed between us. More than thirty years ago we began practice in the same neighborhood of the city, and for a long time were sharp competitors. During that period there was never the slightest misunderstanding between us, but always a state of comradeship and good fellowship. In the difficult cases that fell to our care we assisted each other, lent advice and held discussions for mutual benefit. During this long and close acquaintanceship I had reason to sound the depth of Dr. Frederick's character, and I am able to testify to the largeness and fineness of his nature. He was singularly fair minded and free from prejudices. He had sound judgment, unusual intelligence, and these added to a generous heart made him a successful physician and surgeon, and a loved companion. His painful illness and most regrettable demise have aroused in me a multitude of recollections. I should have liked to recount to the Academy this evening some reminiscences of his early professional career, some of his experiences which are known to me peculiarly. I recall the days when neither of us could afford to keep a horse, and when our armamentarium was interchangeable and even then incomplete. But what Frederick lacked in means at that time he more than made up by his professional enthusiasm and his devotion to the highest ideals. I shall always revere his memory and continue to think of him with loyalty and affection.

Very respectfully,
CHARLES G. STOCKTON.

